# Utility of the amplitude of RV_1_+SV_5/6_ in assessment of pulmonary hypertension

**DOI:** 10.1371/journal.pone.0206856

**Published:** 2018-11-26

**Authors:** Sachiyo Igata, Nobuhiro Tahara, Yoichi Sugiyama, Munehisa Bekki, Jun Kumanomido, Atsuko Tahara, Akihiro Honda, Shoko Maeda, Kazutaka Nashiki, Tomohisa Nakamura, Jiahui Sun, Toshi Abe, Yoshihiro Fukumoto

**Affiliations:** 1 Division of Cardiovascular Medicine, Department of Medicine, Kurume University School of Medicine, Kurume, Japan; 2 Department of Radiology and Center for Diagnostic Imaging, Kurume University School of Medicine, Kurume, Japan; Keio University, JAPAN

## Abstract

Electrocardiogram (ECG) has been widely used for assessment of right ventricular (RV) hypertrophy (RVH) in patients with pulmonary hypertension (PH). However, it still remains unclear which ECG criteria of RVH are useful to predict for the severity of PH. The aim of our study was to examine the utility of ECG findings of RVH in assessment of PH. A total of 53 patients (42 women, mean age; 57.6 ± 16.4 years) with pre-capillary PH, who were diagnosed by right heart catheterization, underwent blood sampling, ECG, and cardiac magnetic resonance within a week before the right heart catheterization. We assessed the traditional ECG criteria of RVH in PH patients, and compared to age- and gender-matched control subjects without PH confirmed by 2-dimensional echocardiography (n = 42, mean age 55.3 ± 15.9 years). We also analyzed the clinical variables associated with ECG findings in patients with PH. Mean pulmonary arterial pressure (mPAP), cardiac index, and pulmonary vascular resistance (PVR) in PH patients were 35.3 ± 11.9 mmHg, 2.82 (2.09–3.45) L/min/m^2^, and 576 ± 376 dyne·sec·cm^-5^, respectively. The prevalence of right axis deviation (43.4%), R:S ratio V_1_ > 1 (32.1%), and RV_1_+SV_5/6_ > 10.5 mm (69.8%) in PH patients was greater than those in control subjects (p < 0.001). In univariate analysis, mPAP, PVR, RV wall thickness, RV mass index, RV volume, and RV ejection fraction (EF) (inversely) were significantly correlated with the amplitude of RV_1_+SV_5/6_. Multiple regression analysis revealed that mPAP and RVEF (inversely) were independently associated with the amplitude of RV_1_+SV_5/6_ (R^2^ = 0.282). Also, we performed the survival analysis among pre-capillary PH patients. During a mean follow-up of 3.7 years, patients with ≥ 16.4 mm of RV_1_+SV_5/6_ had worse prognosis than those with < 16.4 mm (Log rank p = 0.015). In conclusion, the amplitude of SV_1_+RV_5/6_ could be the most useful factor reflected for RV remodeling, hemodynamics and survival in patients with pre-capillary PH.

## Introduction

Pre-capillary pulmonary hypertension (PH) is a progressive disease characterized by increased pulmonary vascular resistance (PVR), which causes right ventricular (RV) remodeling such as hypertrophy and/or enlargement [1, 2], ultimately resulting in right heart failure and death [3, 4]. Therefore, accurate assessment of RV remodeling is important to evaluate the disease severity in patients with pre-capillary PH.

Although a 10-year follow-up study in patients with pre-capillary PH demonstrated that patients with mean PAP (mPAP) ≥ 42.5 mmHg showed worse survival rates than those with mPAP < 42.5mmHg [5], patients with pre-capillary PH can present the different courses of RV remodeling according to the disease severity; one is adaptive remodeling, and the other is maladaptive. RV hypertrophy (RVH) is initially an adaptive physiological response to increased overload. Adaptive remodeling is characterized by the increased RV wall thickness/mass and the preserved RV function, whereas maladaptation is related to the enlarged RV and the reduced RV function [6]. If the overload persistently continues, adaptive remodeling transitions to maladaptive remodeling. RV function has a significant impact on the prognosis of PH [7]. Reduced RV ejection fraction (RVEF) less than 25–35% is a prognostic factor for worse outcome among the PH patients [8, 9]. Especially, RVH is one of the triggers of RV dysfunction in PH. Although echocardiography and cardiovascular magnetic resonance (CMR) imaging are established for assessment of the RVH and RV function [10, 11], a low-cost equipment is required.

Guidelines from the American Heart Association, the American College of Cardiology Foundation and the Heart Rhythm Society had indicated ECG criteria for diagnosis of RVH [12]. Although ECG criteria have been widely used for screening of RVH, they often remain challenging to precisely evaluate RV remodeling in PH [13, 14]. Though there are many ECG studies on PH, ECG criteria of RVH have low sensitivity and low specificity for diagnosis of PH [12, 15]. The 2015 European Society of Cardiology/European Respiratory Society guidelines recommend the utility of right heart catheterization (RHC) and CMR for diagnosis and severity of PH [3]. However, the association between ECG findings and clinical variables including both RHC and CMR in PH are not fully investigated yet. Also, it is unknown whether the ECG parameter in PH patients can be a prognostic factor. Therefore, in the current study, we aimed to examine the utility of ECG findings of RVH in assessment of PH.

## Materials and methods

### Participants

This study included 53 consecutive patients with pre-capillary PH, who were diagnosed by right heart catheterization in Kurume University Hospital from January 2013 to February 2016. Pre-capillary PH was defined as a mPAP ≥ 25 mmHg, pulmonary arterial wedge pressure (PAWP) ≤ 15 mmHg, and PVR ≥ 240 dyne·sec·cm^-5^ at rest. All patients underwent blood sampling, ECG, and CMR within a week before the right heart catheterization. The endpoint for survival analyses was defined as all cause of death until July 2018. During a follow-up period, lung transplantation and death were defined as all cause of death. Forty-two age- and gender-matched control subjects without PH confirmed by 2-dimensional echocardiography, who received blood sampling and ECG due to non-fatal arrhythmias, were also enrolled. The study was conducted in accordance with ethics guidelines introduced by the Declaration of Helsinki and was approved by the Ethics Committee of Kurume University. All subjects provided written informed consent.

### Electrocardiography

A 12-lead ECG (10 mm = 1 mV, 25 mm/s) was acquired in a supine position during quiet respiration (ECG-1550; NIHON KOHDEN, Fukuoka, Japan). ECG findings including heart rate, frontal QRS axis, and amplitude of P, R, and S waves were assessed. Amplitude of P, R, and S waves was averaged from 3 consecutive cardiac cycles. The interpreter for ECG findings was blind to patients’ clinical information. We decided 10 ECG criteria of RVH based on the 2009 AHA/ACCF/HRS guideline and Murphy’s study [12, 15].

### Hemodynamic measurements

The diagnosis of pre-capillary PH was confirmed by hemodynamic evaluation with right heart catheterization at rest. Hemodynamic measurements were performed with a Swan-Ganz catheter (Baxter Healthcare Corporation, Santa Ana, CA, USA) in the recumbent position. Cardiac output (CO) was determined using the Fick’s method. The cardiac index (CI) was derived by normalization of CO with the body surface area (BSA): CI = CO/BSA. PVR was calculated from the transpulmonary gradient and CO: PVR = 80 × [mPAP–PAWP] ÷ CO.

### Blood sampling

After overnight fast, peripheral blood was drawn from the antecubital vein for measurements of blood cell counts, lipid profiles, liver and renal function markers, glycemic parameters, uric acid, and N-terminal pro-brain natriuretic peptide (NT-pro-BNP). These chemistries were measured at a commercially available laboratory in Kurume University Hospital.

### CMR imaging

ECG-gated CMR imaging was performed with a standardized clinical protocol on a 3.0-T system (MAGNETOM Skyra; Siemens, Erlangen, Germany). To quantify ventricular end-systolic volume, end-diastolic volume, stroke volume, mass index and ejection fraction (EF), two experienced radiologists semi-automatically traced the ventricular endocardial and epicardial contours in the end-systolic and end-diastolic frames of transaxial slices using dedicated software. Cardiac volume was corrected for BSA.

### Echocardiography

Echocardiogram was performed using the commercially available ultrasound units, General Electric Vivid 7 (GE Medical Systems, Milwaukee, WI) by Japanese registered sonographer. All echocardiographic parameters were calculated according to the American society of echocardiography guideline [16].

### Statistical analysis

Data were presented as mean ± standard deviation or medians with the interquartile range. The Shapiro-Wilk test was performed to evaluate the assumption of normality. Statistical analysis was performed by means of appropriate parametric and nonparametric methods. Unpaired Student t test was performed for comparisons between PH patients and control subjects. Chi-square test was used for categorical variables. Pearson correlations were used to compare between ECG findings and clinical factors. The determinants for RVH criteria were identified by multivariate regression analysis. Receiver-operator characteristics plotting was performed to identify the cut-off value of RV_1_+SV_5/6_ or RV_1_ for severe PH with mPAP ≥ 42.5 mmHg and/or RVEF < 35%, which are associated with poor outcome. Survival analyses were performed using the Kaplan-Meier method and the log-rank test. The relationship between survival and selected variables was analyzed with the Cox proportional hazards model for all-cause mortality adjusted by the age and gender. Values of p < 0.05 were considered to indicate statistical significance. All statistical analyses were performed with the use of the SPSS system (IBM, Chicago, IL, USA).

## Results

### Patient characteristics

Table 1 presents clinical characteristics of 53 patients with pre-capillary PH and 42 control subjects. Thirty-five (66.0%) with pulmonary arterial hypertension (PAH), 12 (22.6%) with PAH coexisting pulmonary disease, and 5 (9.4%) with CTEPH patients were enrolled in the present study. The mean age was 57.6 ± 16.4 years and female predominance (79.2%) was observed in PH patients. Patients with pre-capillary PH had been treated with home oxygen therapy [26 (49.1%)], anticoagulation therapy [27 (50.9%)], diuretics [23 (43.4%)], and/or PH specific therapies consisting of prostacyclin analogs [15 (28.3%)], phosphodiesterase type 5 inhibitors (PDE5-Is) [21 (39.6%)], endothelin receptor antagonists (ERAs) [21 (39.6%)], and soluble guanylate cyclase stimulators [1 (1.9%)]. Under these treatments, WHO functional classification was predominantly class II (26.4%) and class III (62.3%). Systolic pulmonary artery pressure (sPAP), mPAP, cardiac index, PVR, and mean right atrial pressure were presented with 57.6 ± 20.8 mmHg, 35.3 ± 11.9 mmHg, 2.82 (2.09–3.45) L/min/m^2^, 576 ± 376 dyne·sec·cm^-5^, and 5.0 (3.0–8.0) mmHg, respectively. CMR revealed RV free wall thickness of 3.7 (3.1–4.4) mm and RVEF of 36.9 ± 9.6%. LV wall thickness and LVEF were not significantly different in both groups (p > 0.05).

**Table 1:** Characteristics of study population.

Parameters	Control subjects	Patients with pre-capillary PH	p-value
Number	42	53	
Age	55.3 ± 15.9	57.6 ± 16.4	0.493
Female, n (%)	29 (69.0%)	42 (79.2%)	0.256
Cause of PH			
I/HPAH	-	12 (22.6%)	
CTD-PAH	-	15 (28.3%)	
CHD-PAH	-	4 (7.5%)	
Pulmonary disease	-	12 (22.6%)	
CTEPH	-	5 (9.4%)	
Others	-	5 (9.4%)	
Systolic blood pressure, mmHg	128.8 ± 22.8	118.7 ± 21.0	**0.030**
Heart rate, bpm	65.9 ± 10.6	77.5 ± 14.8	**< 0.001**
Pulmonary hemodynamics			
sPAP, mmHg	-	57.6 ± 20.8	
mPAP, mmHg	-	35.3 ± 11.9	
PVR, dyne·sec·cm^-5^	-	576 ± 376	
CI^#^, L/min/m^2^	-	2.82 (2.09–3.45)	
mRAP, mmHg	-	5.0 (3.0–8.0)	
6MWD, m	-	374 ± 142	
CMR			
RVEDVI ^#^, mL/m^2^	-	63.9 (51.8–93.5)	
RVESVI ^#^, mL/m^2^	-	38.7 (29.0–55.2)	
RVMI^#^, g/m^2^	-	36.5 (27.9–41.8)	
RVEF, %	-	36.9 ± 9.6	
RVWT^#^, mm	-	3.7 (3.1–4.4)	
LVEDVI^#^, mL/m^2^	-	51.0 (41.8–64.8)	
LVESVI ^#^, mL/m^2^	-	22.1 (17.7–30.1)	
LVMI, g/m^2^	-	51.6 ± 13.9	
Echocardiographic data			
IVST, mm	8.9 ± 1.2	8.4 ± 1.5	0.101
PWT, mm	8.9 ± 1.2	8.6 ± 1.1	0.163
LVDd, mm	44.6 ± 4.4	41.8 ± 6.5	**0.013**
LVDs, mm	27.6 ± 3.7	25.5 ± 4.6	**0.017**
LVEF, %	68.3 ± 5.8	69.4 ± 7.1	0.409
NT-pro-BNP^#^, pg/mL	60.9 (28.9–102.7)	219.3 (71.2–1233.4)	**< 0.001**
Uric acid, mg/dL	4.7 ± 1.3	5.8 ± 2.1	**0.003**
eGFR, mL/min/1.73m^2^	85.6 ± 19.6	80.6 ± 32.2	0.376
Comorbidity, n (%)			
Diabetes mellitus	2 (4.8%)	8 (15.1%)	0.177
Hypertension	15 (35.7%)	15 (28.3%)	0.508
Dyslipidemia	11 (26.2%)	16 (30.2%)	0.819

Values are number (%), mean ± SD, or

^#^median (interquartile range).

Bold indicates statistically significant data.

n, number; IPAH, idiopathic pulmonary arterial hypertension; HPAH, heritable pulmonary arterial hypertension; CTD-PAH, pulmonary arterial hypertension associated with connective tissue disease; CHD-PAH, pulmonary arterial hypertension associated with congenital heart disease; CTEPH, chronic thromoboembolic pulmonary hypertension; sPAP, systolic pulmonary arterial pressure; mPAP, mean pulmonary arterial pressure; PVR, pulmonary vascular resistance; CI, cardiac index; 6MWD, 6-minute walk distance; CMR, cardiovascular magnetic resonance; RVEDVI, right ventricular end-diastolic volume index; RVESVI, right ventricular end-systolic volume index; RVMI, right ventricular mass index; RVEF, right ventricular ejection fraction; RVWT, right ventricular free wall thickness; LVEDVI, left ventricular end-diastolic volume index; LVESVI, left ventricular end-systolic volume index; LVMI, left ventricular mass index; IVST, interventricular septum thickness; PWT, posterior wall thickness; LVDd, left ventricular end-diastolic dimension; LVDs, left ventricular end-systolic dimension; LVEF, left ventricular ejection fraction; NT-pro-BNP, N-terminal pro-brain natriuretic peptide; eGFR, estimated glomerular filtration rate.

### ECG findings

All PH patients and control subjects were in sinus rhythm with an average heart rate of 72.4 ± 14.3 beats/min. Patients with pre-capillary PH had a significantly greater prevalence of high R wave amplitude in lead V_1_, deep S wave amplitude in lead V_5_, high R:S ratio in lead V_1_, low R:S ratio in lead V_5_, right axis deviation, and high R wave amplitude in lead aVR than control subjects (Table 2).

**Table 2:** ECG findings of control subjects and pre-capillary PH patients.

ECG findings	Control subjects	Patients with pre-capillary PH	p-value
RV_1_^#^, mm	2.3 (1.5–3.6)	3.7 (2.1–8.9)	**0.013**
RV_5_^#^, mm	14.9 (11.5–18.2)	11.8 (8.4–16.8)	**0.008**
SV_1_^#^, mm	8.9 (6.5–12.8)	5.0 (2.1–6.8)	**< 0.001**
SV_5_^#^,mm	3.3 (1.5–4.8)	7.1 (4.8–10.6)	**< 0.001**
V_1_ R:S ratio	0.3 ± 0.2	3.8 ± 13.9	**0.014**
V_5_ R:S ratio	6.6 ± 6.3	2.3 ± 2.2	**< 0.001**
RV_1_+SV_5/6_^#^, mm	5.7 (4.2–8.4)	11.5 (8.2–18.2)	**< 0.001**
QRS axis	45.6 ± 30.2	81.1 ± 43.9	**< 0.001**
R in aVR, mm	1.0 ± 0.8	1.6 ± 1.6	**0.010**
P in lead II, mm	1.2 ± 0.4	1.4 ± 68 .4	0.395

Values are number (%), mean ± SD, or

^#^median (interquartile range).

Bold indicates statistically significant data.

Table 3 shows the prevalence of ECG criteria for RVH in PH patients and control subjects. RVH criteria such as RV_1_+SV_5/6_ ≥ 10.5 mm, SV_5_ ≥ 7 mm, QRS axis ≥ 90°, and RV_1_ ≥ 7 mm were frequently observed [37 (69.8%), 28 (52.8%), 23 (43.4%), and 18 (34.0%), respectively] in our PH patients. However, each ECG criterion of RVH was not enough to diagnose the RVH in pre-capillary PH. Of all ECG changes of RVH, the amplitude of R wave in lead V_1_ plus S wave in lead V_5/6_ was the most frequent finding in our PH patients.

**Table 3:** Traditional ECG criteria of RVH in control subjects and with pre-capillary PH patients.

ECG criteria of RVH	Control subjects	Patients with pre-capillary PH	p-value
RV_1_ ≧ 7 mm	4 (9.5%)	18 (34.0%)	**0.005**
SV_5_ > 7 mm	4 (9.5%)	28 (52.8%)	**< 0.001**
RV_5_ < 5 mm	0 (0%)	1 (1.9%)	0.371
RV_1_+SV_5/6_ > 10.5 mm	6 (14.3%)	37 (69.8%)	**< 0.001**
V_1_ R:S > 1	0 (0%)	17 (32.1%)	**< 0.001**
V_5_ R:S < 1	0 (0%)	11 (20.8%)	**0.002**
V_1_ R:S > 1 with R > 5 mm	0 (0%)	11 (20.8%)	**0.002**
SV_1_ ≦ 2mm with RV_5_ ≧ 4 mm	0 (0%)	12 (22.6%)	**< 0.001**
QRS axis > 90°	1 (2.4%)	23 (43.4%)	**< 0.001**
P in lead II > 2.5 mm	0 (0%)	3 (5.7%)	0.117

Values are number (%).

Bold indicates statistically significant data.

### Determinants of clinical variables for ECG findings

**Fig 1:**
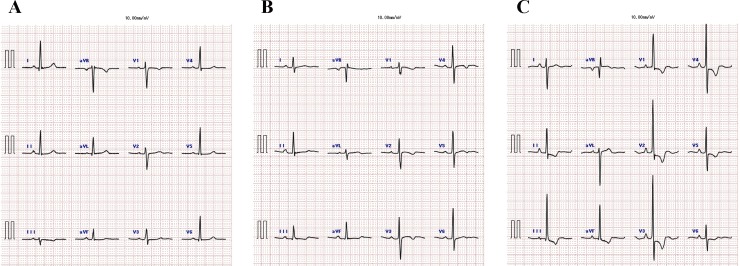
Representative electrocardiogram traces obtained from a control subject without pulmonary hypertension (PH). (A), and patients with mild PH (B) and severe PH (C).

**Fig 2:**
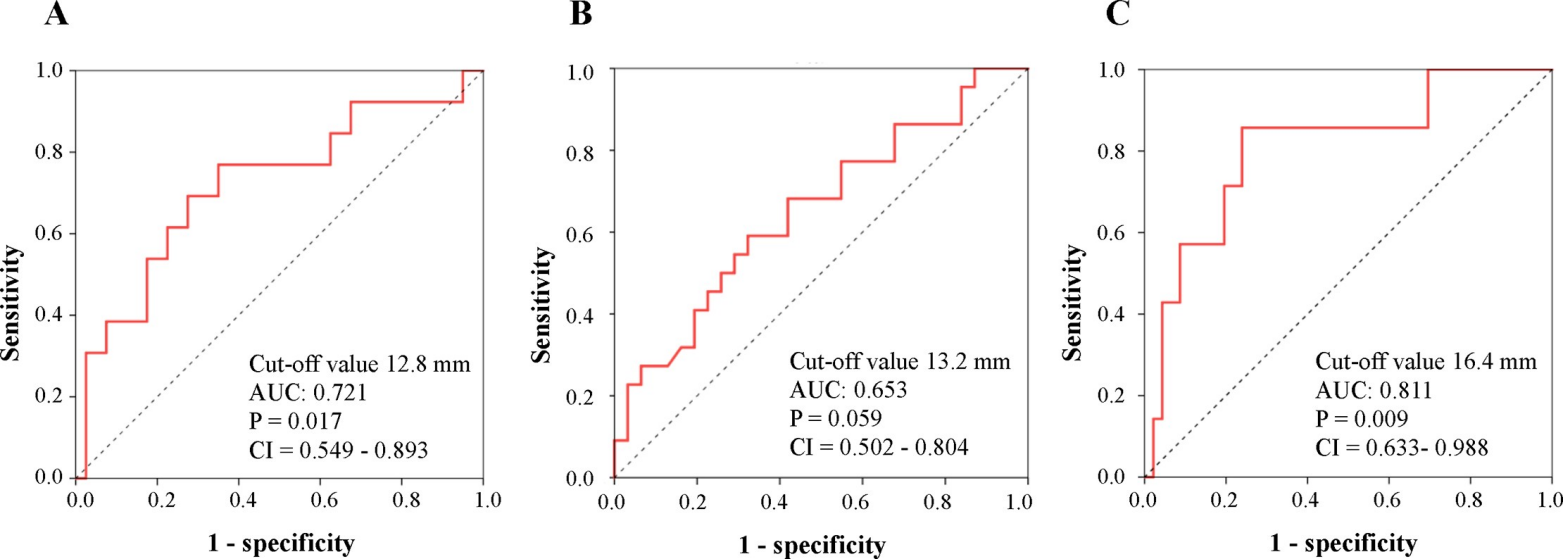
Receiver operating characteristic plottings of the RV_1_+SV_5/6_ for predicting the severity of PH. mPAP ≥ 42.5 mmHg (A), RVEF < 35% (B) and mPAP ≥ 42.5 mmHg + RVEF < 35% (C). AUC, area under curve; CI, confidence interval.

**Fig 3:**
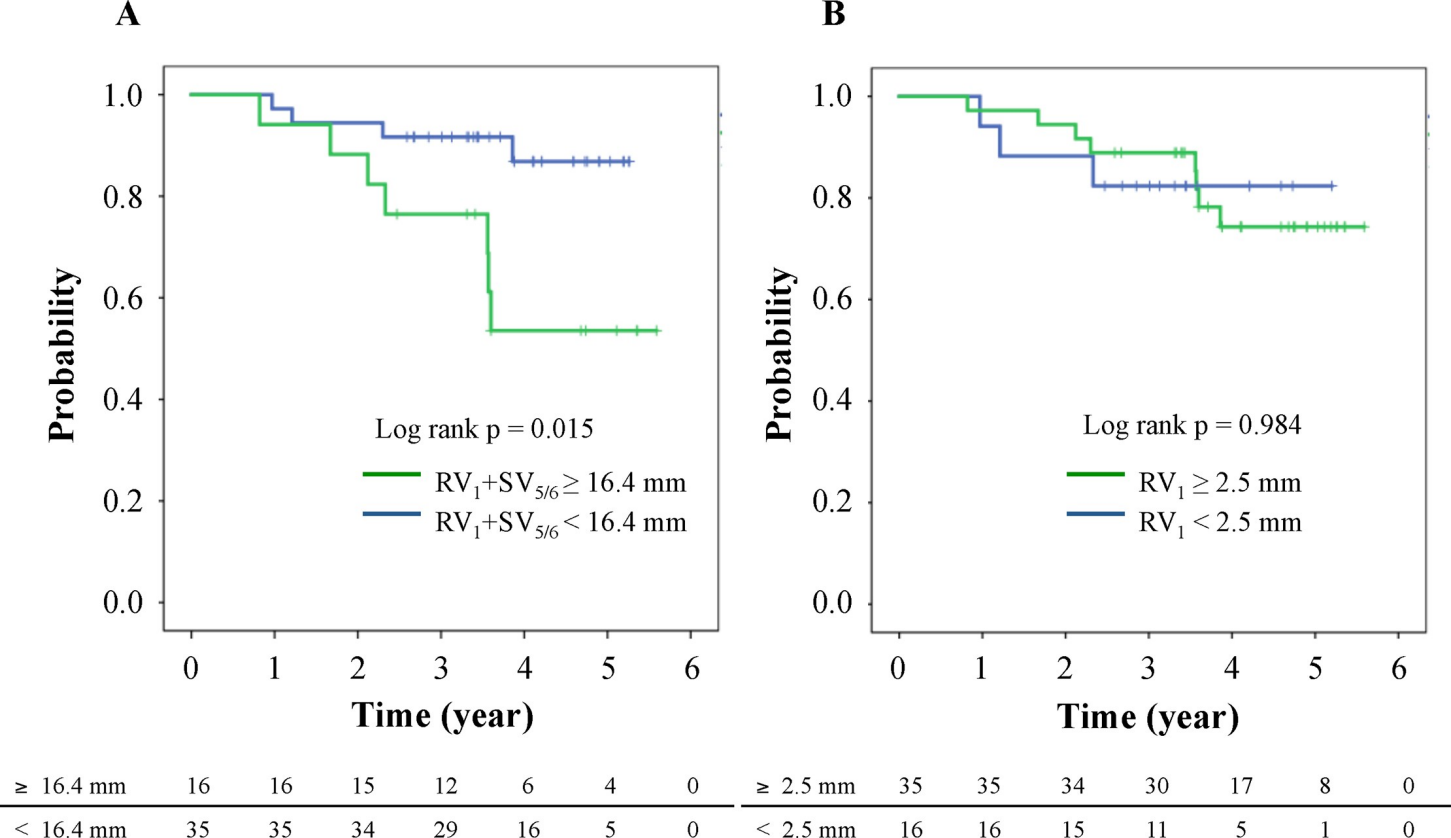
Kaplan-Meier Curves for all cause mortality in patients with pre-capillary PH. RV_1_+SV_5/6_ (A) and RV_1_ (B).

Table 4 shows correlations between the clinical variables and ECG parameters. The QRS axis was significantly correlated with NT-pro-BNP (r = 0.316, p < 0.05). There was no significant correlation between uric acid and ECG findings. In univariate analyses, the amplitude of R in lead V_1_, and RV_1_ + SV_5/6_, R in lead aVR, or QRS axis was significantly correlated with pulmonary hemodynamics (p < 0.01). Also, the amplitude of R in lead V_1_ or RV_1_+SV_5/6_ was correlated with CMR parameters including RV free wall thickness (RVWT) and RV mass index (RVMI) (p < 0.05), RV end-systolic volume index (ESVI) (p < 0.05), RV end-diastolic volume index (EDVI) (p < 0.05), RVEDVI/left ventricular EDVI (p < 0.05), and RVEF (inversely, p < 0.05). Because these significant parameters could be closely correlated with each other, we performed multiple stepwise regression analyses to determine independent associates of the amplitude of RV_1_+SV_5/6_. Multiple regression analyses revealed that mPAP (p = 0.002) and RVEF (p = 0.007) were independently associated with the amplitude of RV_1_+SV_5/6_ (R^2^ = 0.282) (Table 5). Fig 1 shows representative ECG traces in a control subject without PH, and patients with mild PH and severe PH. To seek the cut-off value of RV_1_+RV_5/6_ for severe PH, we performed ROC analysis and compared criteria for severe PH (mPAP ≥ 42.5 mmHg and/or RVEF < 35%) (Fig 2A–2C). The largest area under curve (AUC) of the ROC curve was shown in combination with mPAP ≥ 42.5 mmHg and RVEF < 35% (AUC: 0.811, confidence interval 0.633–0.988, p = 0.009), and defined the cut-off value of 16.4 mm in RV_1_+SV_5/6_ (sensitivity 85.7%, specificity 76.1%) (Fig 2C). We examined all-cause mortality in our patients with pre-capillary PH. During a mean follow-up of 3.7 years, patients with ≥ 16.4 mm of RV_1_+SV_5/6_ had worse prognosis than those with < 16.4 mm (log-rank test, p = 0.015) (Fig 3A). No significant difference of survival was observed between the low amplitude of RV_1_ and the high one (Fig 3B). Moreover, the high amplitude of RV_1_+SV_5/6_ was significantly associated with all cause of death in the Cox proportional hazards regression model with adjustment for age and gender (p = 0.044) (Table 6). There was no lung transplantation in this study population.

**Table 4:** Correlations between clinical parameters and ECG findings.

	RV_1_^#^	RV_5_^#^	SV_1_^#^	SV_5_^#^	R/S V_1_	R/S V_5_	RV_1_+SV_5/6_^#^	QRS axis	R in aVR	P in lead II
sPAP	.406**	-.099	-.288*	.257	.229	-.201	.511**	.459**	.376**	.026
mPAP	.338*	-.153	-.312*	.247	.208	-.226	.446**	.469**	.423**	.075
PVR	.231	-.371**	-.330*	.314*	.262	-.349*	.409**	.514**	.478**	.202
CI^#^	-.096	.345*	.076	-.085	-.161	.189	-.082	-.263	-.309*	-.085
RVWT^#^	.297*	-.117	.009	.072	.229	.067	.411**	.369**	.192	.132
RVEDVI^#^	.316*	.153	-.065	.222	-.038	-.198	.403**	.242	.100	.029
RVESVI^#^	.347*	.109	-.101	.223	.006	-.199	.472**	.192	.111	.026
RVMI^#^	.383*	.205	-.090	.203	.162	-.028	.552**	.307*	.228	-.055
RVEF	-.272*	.041	.119	-.064	-.122	.034	-.407**	.068	-.074	.016
RVEDV/LVEDV	.303*	-.119	-.081	.103	-.047	-.159	.311*	.196	.048	.107
6MWD	.147	.220	-.098	-.137	.109	.081	-.032	-.177	.179	-.119
NT-pro-BNP^#^	-.170	-.098	.093	.248	-.040	-.144	.158	.316*	-.128	.013
Uric acid	.028	-.050	.138	.018	-.154	-.009	.016	.211	-.025	-.038

^#^Log-transformed value was used.

Values are regression coefficients.

*p < 0.05

**p < 0.01

Abbreviations as in Table 1.

**Table 5:** Univariate and multiple stepwise regression analyses for associates of the amplitude of RV_1_+SV_5/6_.

Parameters	Univariate		Multivariate	
	β	p-value	β	p-value
mPAP	0.446	**0.001**	0.385	**0.002**
PVR	0.409	**0.002**	-	-
RVWT^#^	0.411	**0.002**	-	-
RVEF	-0.407	**0.002**	-0.338	**0.007**
RVEDVI/LVEDVI	0.311	**0.024**	-	**-**
R^2^	-		0.282	

^#^Log-transformed value was used.

Bold indicates statistically significant data.

Abbreviations as in Table 1.

**Table 6:** Cox hazard regression analysis for all cause of death.

Parameters	Univariate		
	HR	95% Cl	p-value
sPAP	1.035	0.997–1.074	0.069
mPAP	1.046	0.987–1.110	0.131
PVR	1.151	1.013–1.309	**0.031**
CI^#^	0.133	0.009–2.005	0.145
RVWT	1.526	1.018–2.285	**0.040**
RVEDVI^#^	2.695	0.764–9.511	0.123
RVESVI^#^	2.572	0.954–6.937	0.062
RVMI^#^	8.920	1.357–58.654	**0.023**
RVEF	0.954	0.904–1.007	0.087
LVEF	0.989	0.926–1.057	0.744
6MWD	0.993	0.986–1.000	**0.048**
NT-pro-BNP^#^	1.467	1.014–2.121	**0.042**
Uric acid	1.357	0.997–1.847	0.052
RV_1_+SV_5/6_^#^	3.718	1.038–13.313	**0.044**
RV_1_^#^	1.252	0.629–2.492	0.522

^#^Log-transformed value was used.

Bold indicates statistically significant data.

HR, hazard ratio; 95% CI, 95% confidence interval.

Other abbreviations as in Table 1.

## Discussion

In this study, we examined the utility of ECG findings of RVH in the assessment of pre-capillary PH. Our study demonstrated that the amplitude of RV_1_+SV_5/6_ was the most frequent finding as a traditional ECG criterion of RVH. Further, mPAP and RVEF was independently associated with the amplitude of RV_1_+SV_5/6_. This is the first study to examine the association between ECG findings and the clinical variables evaluated by both RHC and CMR, including ECG parameter as a prognostic factor. A significant finding was that RV_1_+SV_5/6_ is a powerful predictor of prognostic factors; cut-off value of over 16.4 mm in RV_1_+SV_5/6_ derived from our study could predict the worse prognosis in patients with pre-capillary PH.

Pre-capillary PH is diagnosed by the standard right heart catheterization [3]. RV volume, RVEF and RV free wall thickness can be accurately assessed by CMR [17]. Thus, we assessed pulmonary hemodynamics by right heart catheterization and RV remodeling by CMR. However, it is difficult for outpatients to constantly perform these examinations within follow-up periods. Non-invasive, low-cost ECG can be repeatedly performed in patients with PH. Indeed, traditional ECG criteria have been used for screening of RVH in clinical practice [12]. The relation between traditional ECG criteria of RVH and PH has been published previously [13, 14, 18, 19]. The sensitivity and specificity for RVH differently depends on the study subjects. In particular, ECG criteria of RVH were determined from patients with congenital heart disease (CHD) [12, 20]. Whereas CHD such as atrial septal defect yields the right heart volume overload, pre-capillary PH shows the RV pressure overload. Therefore, it is difficult to divide RVH and RV enlargement using ECG criteria of RVH.

Pre-capillary PH is characterized by elevated PVR leading to RV pressure overload, which leads to RV deformity including ventricular hypertrophy and/or enlargement [3, 21, 22]. RV enlargement (RVEDVI ≥ 84 mL/m^2^) and reduced stroke volume (SVI ≤ 25 mL/m^2^) have been shown as poor prognostic factors in patients with pulmonary arterial hypertension (PAH). Moreover, heart failure and reduced RVEF in PH patients decrease survival rate [21]. Both mPAP ≥ 42.5 mmHg and RVEF < 35% are predictive factors for poor outcome in patients with PH [5, 8]. Therefore, assessment of the hemodynamics, RV structure, and RV function are essential for prediction of prognosis in patients with PH. When we sought the non-invasive ECG criteria associated with pulmonary hemodynamics and RV remodeling in pre-capillary PH, the amplitude of RV_1_+SV_5/6_ was the most frequent ECG criterion of RVH. Further, mPAP and RVEF was independently associated with the amplitude of RV_1_+SV_5/6_. Obviously, RVEDV and RVESV leading to RVEF were correlated with the amplitude of RV_1_+SV_5/6_. Moreover, pulmonary hemodynamic parameters were related to RVEDVI and RVESVI (p < 0.01). RVEF was negatively correlated with PVR (r = -0.296, p < 0.05). These findings suggest that the amplitude of RV_1_+SV_5/6_ may be a useful variable associated with hemodynamics and RV remodeling in patients with pre-capillary PH.

Kopec et al. examined the ECG criteria for predicting RVH and increased RV volume, RV_1_+SV_5/6_ had a correlation with the RV mass index (RVMI: r = 0.54, p = 0.008) but not RV volume (r = 0.05, p = 0.82), RV_1_+SV_5/6_ had a significant AUC with high sensitivity and specificity for predicting the RVH (AUC = 0.78, p = 0.03, sensitivity 81%, specificity 57%) [19]. In our study, RV_1_+SV_5/6_ had a correlation with both RVMI and RV volume (RVMI: r = 0.552, p < 0.001, RVEDVI: r = 0.403, p = 0.003, RVESVI: r = 0.472, p < 0.001). While diagnostic accuracy of RV_1_+SV_5/6_ for RVH was lower than Kopec’s study (AUC = 0.697, sensitivity 54.5%, specificity 88.9%, the cut-off value of RV_1_+SV_5/6_: 12.7 mm), RV_1_+SV_5/6_ predicted the worse prognosis with mPAP ≥ 42.5 mmHg and/or RVEF < 35%.

The amplitude of R in lead V_1_ characterized by RV pressure overload is widely used for assessment of the RVH severity. It is well known that increased R amplitude in lead V_1_ is common in adolescent subjects. Therefore, we compared the electrocardiographic RVH parameters between patients with PH and age-matched control subjects, and found that the R amplitude in lead V_1_ was greater in PH patients than in controls. It has shown that the amplitude of R in lead V_1_ is correlated with RV mass index by CMR [19]. Cheng XL et al. reported that the amplitude of RV_1_ or SV_6_ was correlated with mPAP [23]. Sato S et al. showed that decrease in RV_1_ predicted the better survival in patients with PAH [24]. As with the Cheng’s study, the amplitude of RV_1_ or SV_5/6_ was correlated with mPAP in our study (RV_1_, r = 0.338; SV_5/6_, r = 0.334; p < 0.05, respectively). Also, the amplitude of RV_1_+SV_5/6_ was significantly associated with PAP and CMR parameters including RV size, function, wall thickness and mass (Table 4). The cut-off value of 16.4 mm in RV_1_+SV_5/6_predicted prognostic factors such as mPAP ≥ 42.5 mmHg and RVEF < 35% (AUC 0.811, p = 0.009, sensitivity 85.7%, specificity 76.1%). Although RV_1_ presented a high AUC (AUC 0.795, p = 0.013) similar to RV_1_+SV_5/6_, specificity of the cut-off value of 2.5 mm in RV_1_ was inferior to RV_1_+SV_5/6_ (sensitivity 100%, specificity 37%).

As widely known, ECG changes depend on left ventricular volume [25]. Therefore, we excluded patients with post-capillary secondary PH such as hypertrophic/dilated cardiomyopathy, ischemic heart disease, and valvular disease. In the current study, the amplitudes of RV_1_+SV_5/6_ and RV_1_ were significantly correlated with RVEDVI to LVEDVI ratio (p < 0.05). Ogawa et al reported that mPAP ≥ 42.5 mmHg showed worse survival rate in patients with idiopathic/heritable PAH [5]. Also, RVEF < 35% is associated with a poor outcome regardless of PVR values [8]. In our study, the cut-off value of 16.4 mm in RV_1_+SV_5/6_ predicts the both mPAP ≥ 42.5 mmHg and RVEF < 35%. Also, we performed the survival analysis among pre-capillary PH patients. During a mean follow-up of 3.7 years, patients with ≥ 16.4 mm of RV_1_+SV_5/6_ had worse prognosis than those with < 16.4 mm (Fig 3A). Moreover, the amplitude of RV_1_+SV_5/6_ was significantly associated with all cause of death in the Cox proportional hazards analysis (Table 6). On one hand, the amplitude of RV_1_ did not predict the survival in this study (Fig 3B). Thus, the amplitude of RV_1_+SV_5/6_ could be a predictor for prognosis in patients with pre-capillary PH.

NT-pro-BNP is well recognized as a biomarker of disease severity in post-capillary PH. Also, elevated NT-pro-BNP is associated with worse prognosis in pre-capillary PH [26–30]. We found that NT-pro-BNP was associated with PVR, RV volume, 6MWD, QRS axis, and S amplitude in lead V_5_. The amplitude of RV_1_+SV_5_ might include the factors derived from not only right ventricle, but also left ventricle.

### Study limitations

Several limitations should be mentioned for the present study. First, the small study size limits our interpretation and discussion. Also, our PH patients consisted of different etiologies. It was difficult to enroll many patients with a homogeneous etiology, because PH is a rare disease and has a poor prognosis. A previous study by Nagai et al. demonstrated that ECG findings for RVH predict the presence of RV systolic dysfunction assessed by CMR in patients with pre-capillary PH [31]. Also, Nishiyama et al. reported that therapeutic improvement of ECG findings for RVH after balloon pulmonary angioplasty was correlated to that of hemodynamics in patients with CTEPH [32]. However, there have been no ECG studies assessed for the severity and the prognosis of pre-capillary PH using both CMR and RHC. Second, we did not perform RHC and CMR in control subjects. In the study, all control subjects were confirmed without significant organic heart disease by 2-dimensional echocardiography. Third, we did not consider the negative T-wave in the limb and precordial leads in our patients. Negative T wave is relatively common in acute pulmonary embolism, and is not correlated with RV free wall thickness [33]. As T wave change and R amplitude in lead V_1_ depend on age of the patients, we compared our patients with age-matched control subjects. Fourth, low voltage in the limb leads was frequently present in patients with pulmonary disease owing to increased lung volume. There were 12 patients with pulmonary disease in our study. Although we did not include to analyze the amplitude in limb leads except aVR as electrocardiographic RVH parameters in this study, we can not deny that lung volume may affect our results. Finally, our data lack clinical outcomes to support a utility of ECG findings of RVH in patients with pre-capillary PH. Accordingly, further longitudinal studies are needed to clarify whether the ECG findings of RVH are useful to predict clinical outcomes.

## Conclusion

In conclusion, our study demonstrated that the amplitude of SV_1_+RV_5/6_ could be the most useful factor reflected for RV remodeling, hemodynamics and survival in patients with pre-capillary PH.
